# Seroprevalence of *Chlamydia trachomatis* and Associated Factors among Vulnerable Riverine in the Brazilian Amazon

**DOI:** 10.3390/ijerph192315969

**Published:** 2022-11-30

**Authors:** José Jorge da Silva Galvão, Carlos Leonardo Figueiredo Cunha, Ellen Christiane Correa Pinho, David de Jesus da Silva Paiva, Nádile Juliane Costa de Castro, Valéria Gabriele Caldas Nascimento, Wanderson Santiago de Azevedo Junior, Richardson Augusto Rosendo da Silva, Rosimar Neris Martins Feitosa, Antonio Carlos Rosário Vallinoto, Eliã Pinheiro Botelho, Glenda Roberta Oliveira Naiff Ferreira

**Affiliations:** 1 Program of Post-Graduation in Nursing, Federal University of Para, Belem 66075-110, Brazil; 2 Nursing School, Federal University of Para, Belem 66075-110, Brazil; 3 Program of Post-Graduation in Collective Health, Federal University of Rio Grande do Norte, Natal 59078-970, Brazil; 4 Program of Post-Graduation in Biology of Infectious Agents, Federal University of Para, Belem 66075-110, Brazil

**Keywords:** *Chlamydia trachomatis*, sexually transmitted infections, prevalence, primary healthcare, serology

## Abstract

Due to social and individual conditions and access to health services, Amazonian riverside populations are highly vulnerable to sexually transmitted infections, including *Chlamydia trachomatis*. The aim is to estimate the seroprevalence of *Chlamydia trachomatis* and analyze the associated factors among riverside dwellers in a capital city in the Brazilian Amazon. A cross-sectional study was carried out with residents of the Combu Island, Belém. The study sample was calculated using the population survey technique in the EPI INFO. Only people aged 18 and over were included. ELISA serology was performed to detect antibodies against *Chlamydia trachomatis*. For data collection, a form containing vulnerability factor questions was applied. Binary regression analysis was performed using the Minitab 20 program. The study sample consisted of 325 participants. The prevalence of IgG/IgM antibodies against *Chlamydia trachomatis* was 22.2% and 5.5%, respectively. In the multiple regression, only participants with a broken condom were more likely to have antibodies against the bacteria (OR: 1.90; 95% CI: 1.01; 3.37; *p* = 0.046). Seroprevalence was associated with condom breakage. This factor demonstrates that despite having an attitude towards condom use, probably, they may have inadequate knowledge about the correct practice of introduction.

## 1. Introduction

Globally, *Chlamydia trachomatis* remains the most common of the four curable sexually transmitted infections (STIs), with an estimated 128 million new infections in 2020 [1], and rates vary by world region [2]. However, *C. trachomatis* disproportionately affects vulnerable populations. *C. trachomatis* infections lead to critical adverse outcome of infections if left untreated, such as infertility and adverse pregnancy outcomes, mainly in higher in low- and middle-income countries [3,4,5].

Brazil continues to use syndromic management and, due to the gaps of laboratory diagnosis of the infection and of reporting of cases in primary healthcare, the reported case prevalence and incidence data do not represent the national, regional and local burden of infection [4,6]. Among small communities from the Brazilian Amazon, such as riverine, the lack of data is more concerning, due to the asymptomatic nature of some chlamydia infections and complications associated with it [4].

Populations living on the banks of rivers and lakes are called riverine. In the Brazilian Amazon, riverine populations are affected by social inequality with low income and education levels, limited access to the urban area and low adherence to condom use, and health problems are mostly solved with allopathic medicines, creating a situation of vulnerability. These aspects are determining factors for the maintenance and/or the emergence of an infection [2,4,7,8,9,10]. In this scenario, only one study was conducted among riverine from four municipalities (remote from urban) located in Marajó island, which found a seroprevalence of 30.9%, but did not assess whether the primary healthcare system is capable of mitigating factors of vulnerability [4,11].

No such investigation has been carried out in riverine communities from urban areas in Brazil with access to Family Health Strategy (FHS) teams, particularly in the Brazilian Amazon. In our hypothesis, access to primary care will decrease *C. trachomatis* infections. Thus, small peri-urban islands were selected with a population covered by an FHS team and which have recently entered the tourist itinerary as a capital in the Brazilian Amazon [12,13].

In a biome such as the Amazon, seroprevalence study for specific populations is a recommended STI surveillance tool [2], since it allows estimating the distribution of the infection and knowing the most vulnerable populations to direct health planning regarding prevention and control actions, in addition to priority actions for research [14]. This study aimed to estimate the seroprevalence of *C. trachomatis* and associated factors among riverine communities from peri-urban islands in the Brazilian Amazon.

## 2. Materials and Methods

This is a cross-sectional and analytical study, which was carried out in peri-urban islands in Belém, northern Brazil. This city measures 1,059,458 square kilometers, of which 25,811 are rural with a total of 39 islands and 11,294 inhabitants, about one percent of the population of the city. Belém is the capital of the state of Pará and has precarious social and health indicators, with a Municipal Human Development Index of 0.746 and 303,600 inhabitants (20.43%) covered by the FHS [15,16].

**Figure 1:**
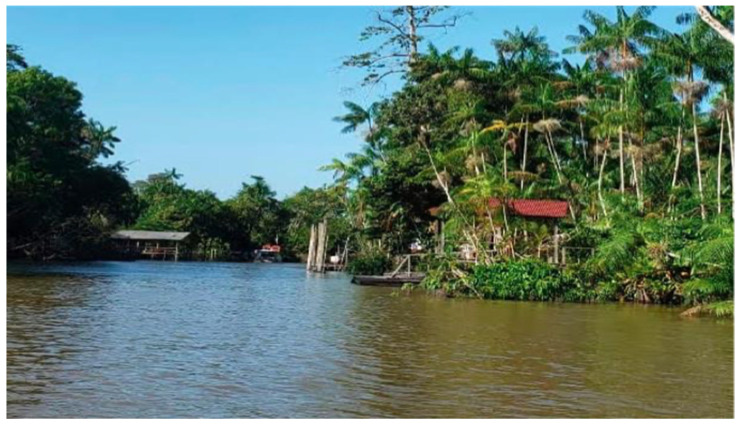
Combú Environmental Protection Area.

The study was carried out on a small island that makes up the Combú Environmental Protection Area (EPA), located in Belém, with about 2200 inhabitants (Figure 1). The river dwellers travel between 15 to 30 min to reach the urban area of Belém; they present low economic status, poor sanitary conditions and geographic characteristics of riverine populations. In recent years, there has been an intense commercial activity focused on tourism on the island, with the presence of bars and restaurants on all the islands of the EPA, and the social facilities are one elementary school, one speedboat for school transport and one Basic Health Unit (BHU) with one FHS team, but there are no speedboats or other means of river transport for FHS teams activities for residents in their places of residence [12,13].

### 2.1. Participants

Data collection took place from August 2020 to January 2021. The target population consisted of residents of the riverside communities of the Combú EPA. Individuals aged 18 years or over were included. Those who did not reside on the islands were excluded.

### 2.2. Variables

The response variable was “serological result *C. trachomatis*”. It was divided into two groups: reagent or non-reagent (dichotomous variable). This variable was treated as a binary dependent variable. The response event selected was a reagent result. The independent variables were the dimensions of individual, social and programmatic vulnerability. Each variable was grouped into one of three dimensions based on the theoretical foundation of vulnerability and on previous studies [17,18,19].

### 2.3. Data Sources

To assess factors of vulnerability, a structured questionnaire adapted from the instrument “knowledge, attitudes and practices in the Brazilian population” [20] was used. It was sent to the macro-project researchers to check for content clarity and adequacy. Its adequacy was established by surveying seven randomly sampled participants who were not included in the study (pre-test). Those who accepted were informed about the objectives, and data were collected by trained nurses (undergraduate students) and graduate students in individual and private face-to-face interviews.

After the interview, the participants were invited to perform a 10 mL blood sample for laboratory tests. The plasma samples were subjected to ELISA assays for the detection of antibodies (Major Outer Membrane Protein). The assays were performed according to the manufacturer’s recommendations. The presence of IgG antibodies against *C. trachomatis* was detected by the Serion Classic *Chlamydia trachomatis* IgG ELISA, Würzburg, Germany, and samples were tested for the presence of IgM antibodies using Serion Classic *Chlamydia trachomatis* IgM ELISA, Würzburg, Germany. The results were provided to the BHU for patient care and treatment, when applicable.

### 2.4. Bias

To reduce selection bias, participants have the same social, economic and health service access characteristics. Therefore, they share the same characteristics that are important predictors of results, being different regarding the demographic composition of sex and age. The recall bias was restricted to some questions regarding access to health services.

### 2.5. Study Size

The sample size and power were calculated using the Statcalc, Epi Info (Version 7.2.2.16) program, according to the following parameters: population size of 2200 adults living in the area of Combú EPA, an acceptable margin of error of five percentage points, a confidence interval of 95%, a design effect of 1.0 and an expected prevalence of *C. trachomatis* of 31% based on previous study [11]. The sample size was 286 participants and a 13% increase for losses, resulting in 323.

**Figure 2:**
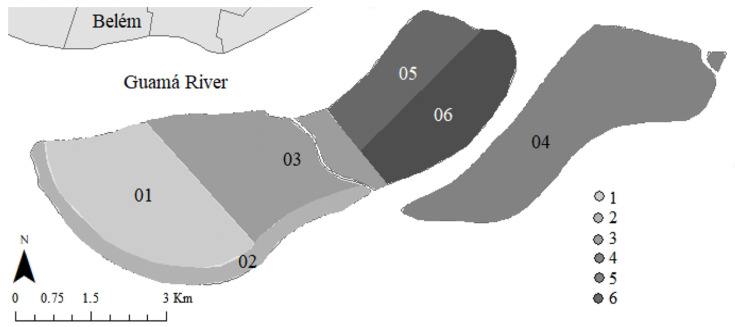
Geographic location of the island investigated in Belém, state of Pará, Brazilian Amazon.

The FHS team provided the researchers with the registration details of individuals aged 18 years or over linked to each of the six micro-areas of the FHS (Figure 2). Then, stratified sampling was carried out proportionally to the number of individuals aged 18 years or over indexed for each micro-area (six strata).

The calculations were performed in the Microsoft Excel^®^ program. Due to the geographic and environmental conditions of the studied area, with flooded areas accessible only by small boats, called “rabetas”, and with a large population dispersion, it was not possible to adopt the random choice of participants. In each of the 18 expeditions carried out, the researchers invited the residents of the different areas of the islands to participate in the study.

### 2.6. Statistical Analysis

Questionnaire information was stored in a database created using the Epi Info 7.2.3 software. For categorical variables, absolute and relative frequencies were calculated. For the continuous variables, measures of central tendency were calculated. The estimate of the prevalence of *C. trachomatis* cases and its confidence interval were calculated by estimating the proportion in the Bioestat 5.3^®^ program. The unanswered questions (missing data) did not have their percentage calculated and were not included in the statistical analysis.

The main hypothesis of the study was that the factors of social, individual, and programmatic vulnerability could predict *C. trachotmatis* infections. This hypothesis was tested using multiple logistic regression in the Minitab 20^®^ software. Firstly, a bivariate regression analysis was used to assess the association between each independent variable and the dependent variable. All variables with a *p* value < 0.20 were entered into a multiple logistic regression equation using a stepwise approach. Crude OR and adjusted odds ratio with their respective 95% CIs were used to evaluate the effect. All *p* values < 0.05 were considered statistically significant. To interpret the results, quality tests, the coefficient value of the independent variable, Z value and 95% CI and OR were considered in the regression.

### 2.7. Ethical Aspects

The invited participants who agreed to participate signed an Informed Consent Form. The study was submitted and approved by the Research Ethics Committee of the Federal University of Pará under protocol No. 3.331.577 and followed the principles of the Declaration of Helsinki.

## 3. Results

The sample of the study comprised 325 participants. The sociodemographic characteristics were: 56.6% (184/325) never attended school or had elementary education; 58.5% were female (190/325); age greater than or equal to 38 represented 47.9% (153/319); 70.2% (228/325) were in a relationship; 70.7% (222/314) had an income below the minimum wage; 74.5% (74.5%; 242/325) of the participants live in a house with three or more people and 66.5% (216/325) are beneficiaries of social programs. As for skin color, most participants (92.8%; 296/319) declared being black, brown or yellow (as classified by the Brazilian Institute of Geography and Statistics) (data shown in Table 1 and Table 2).

The prevalence of antibodies against *C. trachomatis* (IgG/IgM) was 22.2% (72/325; 95% CI: 17.5%; 26.4%). For the isolated IgM marker, the prevalence was 5.5% (6/109; 95% CI: 1.2%; 9.8%) with 100% (06/06) of the cases in females, all with income up to one minimum wage; 1 participant was aged 19 years, and the others were over 30 years old, 3 had secondary level and 3 had fundamental level.

Social factors are shown in Table 1. The beneficiaries of government social programs are almost twice as likely to have reagent serology for *C. trachomatis* (OR: 1.84; 95% CI: 1.01; 3.37; *p* = 0.04). The variables of number of people living in the same house and beneficiaries of government social programs were selected for the multiple regression (*p* < 0.20).

In terms of individual vulnerability (Table 2), participants with broken condoms are twice as likely to have reagent serology for *C. trachomatis* (OR: 2.00; IC: 1.14; 3.51; *p* = 0.01). The variables “condom use” and “sex” and participants with broken condoms (*p* < 20) were selected for the multiple regression.

Table 3 shows the programmatic factors associated with the presence of antibodies against *C. trachomatis*. Participants who did not perform a rapid test for syphilis (OR: 0.55; CI: 0.32; 0.96; *p* = 0.03) and who did not have free access to condoms are less likely to have reagent serology for the studied bacteria (OR: 0.51; CI: 0.30; 0.87; *p* = 0.01). The variables “performed rapid test for Sexually Transmitted Infections—ever in life”, “performed rapid test for HIV—in the last 12 months”, “not performed a rapid test for syphilis” and “not have free access to condoms” were selected for the multiple regression (*p* < 0.20).

The multiple regression model showed that only participants who “had a broken condom” are more likely to have reagent serology for *C. trachomatis* (OR: 1.90; 95% CI: 1.01; 3.37; *p* = 0.046) (Table 4). The other factors showed no significant association after adjustment.

## 4. Discussion

This cross-sectional study estimated the seroprevalence of *C. trachomatis* and associated factors among riverine communities from peri-urban islands in the Brazilian Amazon. Among 325 participants, the prevalence of antibodies (IgG/IgM) for *C. trachomatis* was of 22.2% and the finding of 5.5% was for IgM antibodies. The results show that only one individual factor was associated with *C. trachomatis*. In the final adjusted model, the riverine who had a broken condom were associated with the presence of markers for *C. trachomatis*. In the present study, in the adjusted model, social and programmatic factors together did not minimize this individual vulnerability factor.

The present study found demographic characteristics that confirm the situation of social vulnerability in which the people of the waters of the Amazon live [7]. Unlike other areas of the Amazon, the primary health care service is located in the Combú EPA, reducing barriers to access to the first level of the health system. In other communities, primary care services are located on the mainland in close proximity to communities [8]. For more distant communities, the basic river health unit is a strategy implemented by the Brazilian government [9,21].

In a study that evaluated the seroprevalence of *C. trachomatis* among men from three countries (Brazil, Mexico and the United States) a seroprevalence of 45.5% was found in Brazil [22]. Another study found 63.5% among young US women [3]. In different indigenous populations of the Brazilian Amazon, the seroprevalence reached 50% in some tribes [4]. The study population differs from the populations previously studied, mainly due to the socio-environmental characteristics in which they live. Even located in an urban area, they still retain social, way of life, cultural, infrastructure and environmental aspects that are similar to riverside communities in more remote areas of the Amazon, including the means of transport for the population [7,8,9,12,21]. This study was the first to estimate the prevalence of *C. trachomatis* among riverside communities within an urban center in the Amazon. Thus, the discussion is limited due to the data gap.

The study was carried out with populations that inhabitant municipalities on the island of Marajó and also found the presence of IgM antibodies, with a seroprevalence of 6.7% higher than that found in the study (5.5%) [11], demonstrating the presence of a recent or ongoing infection [4]. Early identification of the bacterium in all populations is important due to the high number of asymptomatic cases that can perpetuate the continuous transmission of the bacterium [3,11,22,23,24].

The univariate analysis of social vulnerability markers found that only receiving benefits from social programs was associated with higher chances of reagent serology for *C. trachomatis*. The riverside populations of the Amazon, despite the environmental wealth, have social conditions that make them vulnerable to injuries and diseases [4,7]. Social benefits are granted to populations with very low or no income, often people residing in areas without social facilities and with low levels of education. In order to reduce social inequities, over the years in Brazil, there has been an expansion of conditional and unconditional income transfer programs and, recently, Emergency Aid was implemented during the COVID-19 pandemic [25,26].

Among the aspects of individual vulnerability, just having a broken condom increased the chances of having serology reactive for antibodies against the bacteria studied. The result of the present study demonstrates the need for the FHS team to carry out prevention actions based on collective and individual health education whose approach and language are understandable for this population. In the programmatic aspect, participants who did not perform a rapid HIV test and those who did not have access to a condom for free in the last 12 months were less likely to have been exposed to *C. trachomatis*. The characteristics of the population studied may explain these results, including the high percentage of participants in a relationship and few sexual partners in the last six months. Previous studies have found an association of the bacteria with having multiple partners and longer sexual activity [3,11,22,23].

Even with an adjusted analysis by multiple binary regression, it was possible to observe that a broken condom was the only factor associated with the presence of anti-*C. trachomatis* antibodies. Previous study demonstrated that only consumption of alcoholic beverages increases the likelihood of being infected with *C. trachomatis* in riverine communities [11].

In Brazil, as a signatory to the World Health Organization, the prevention practices guided by this international body are followed. Initially, health education actions were the first primary prevention measures for STIs that were implemented, mainly with mass media campaigns that stimulated the use of condoms. In recent years, structural and biomedical preventive measures have been incorporated into the Unified Health System [1,6,27,28].

Access to condom use is one of the strategies to eliminate STIs [2]. However, in addition to using the family health strategy, teams should assess the effectiveness of condom use in order to work on collective and individual interventions aimed at the population’s needs [29,30]. It is important that health professionals also advise people on the actions to be taken in case of condom breakage during sexual intercourse to minimize the damage related to a possible infection [31,32].

This cross-sectional study does not make it possible to identify the moment of exposure to the bacteria and the level of antibodies related to the decay that may not be detected by the serological methods of antibody detection. Another limitation is related to the environmental and geographic conditions, due to the collection period in the middle of the Amazonian winter, with heavy rains and the dynamics of the Amazon rivers with high and low times. In this way, the excursions were organized according to these aspects, and it was not possible to access some areas, which prevented the random collection based on the people of the islands. The study only involved one community with access to the FHS team in the Brazilian Amazon. The COVID-19 pandemic period brought limitations caused by periods of greater transmission and morbidity and mortality.

## 5. Conclusions

The prevalence of anti-*C. trachomatis* IgG antibodies was 22.2% in the riverine population. Social and programmatic factors were not associated with serological markers of the bacterium. Only a broken condom during sexual intercourse was associated with the presence of antibodies to the bacteria. It is a factor of individual vulnerability that can be minimized by the work of the FHS team through collective health education actions or during individual consultations with language and means of communication that are understandable by the population of the territory. Knowledge of seroprevalence made it possible to identify the most exposed people among a population at low risk for STIs but who may be at high risk for adverse chlamydia outcomes when untreated.

As contributions, the results bring together advances in knowledge, since they are data from a riverside population with difficult access and with specificities, providing subsidies for the redirection of assistance aimed at the education and empowerment of a community in a context of vulnerability to STIs.

## Figures and Tables

**Table 1:** Social factors associated with serology for *C. trachomatis* among riverine, in the Brazilian Amazon, 2020–2021.

SOCIAL	Non-Reagent	Reagent	Total	Binary Regression		
	*n* (%)	*n* (%)	*n* (%)	Crude OR	95% CI	*p*
Marital status						
Married/stable union/dating	177 (77.6)	51 (22.4)	228 (70.2)	0.95	0.53; 1.70	0.88
Single/separated/divorced/widow(er)	76 (78.4)	21 (21.6)	97 (29.8)		Ref.	
Level of education						
Never attended school/Elementary	142 (77.2)	42 (22.8)	184 (56.6)	1.09	0.64; 1.85	0.73
High school/University	111 (77.6)	30 (22.4)	141 (43.4)		Ref.	
Family income *						
Less than one minimum wage	170 (76.6)	52 (23.4)	222 (70.7)	1.45	0.77; 2.70	0.23
Equal to or greater than 1 minimum wage	76 (82.6)	16 (17.4)	92 (29.3)		Ref.	
NI **	7	4	11			
Skin color						
White	19 (82.6)	4 (17.4)	23 (7.2)			
Black/Brown/yellow	229 (77.4)	67 (22.6)	296 (92.8)	1.38	0.45; 4.22	0.56
NI **	5	1	6			
Number of people living in the house						
Equal to 3 or more	182 (75.2)	60 (24.8)	242 (74.5)	1.95	0.99; 3.84	0.05
1 to 2	71 (85.5)	12 (14.5)	83 (25.5)		Ref.	
Participate in social programs						
Yes	161 (74.5)	55 (25.5)	216 (66.5)	1.84	1.01; 3.37	0.04
No	92 (84.4)	17 (15.6)	109 (33.5)		Ref.	

Legend: * Brazilian monthly minimum wage 2020—BRL 1045.00 per month. ** NI: not informed–not considered for statistical calculation. OR: odds ratio. CI: confidence intervals. Ref.: reference.

**Table 2:** Individual factors associated with serology for *C. trachomatis* among riverine, in the Brazilian Amazon, 2020–2021.

INDIVIDUAL	Non-Reagent	Reagent	Total	Binary Regression		
	*n* (%)	*n* (%)	*n* (%)	Crude OR	*n* (%)	*n* (%)
Sex						
Male	111 (82.2)	24 (17.8)	135 (41.5)	0.63	0.36; 1.10	0.11
Female	142 (74.7)	48 (25.3)	190 (58.5)		Ref.	
Age group (years)						
18 to 27	59 (75.6)	19 (24.4)	78 (24.5)	1.43	0.74; 2.78	0.28
28 to 37	66 (75)	22 (25.0)	88 (27.6)	1.48	0.79; 2.80	0.21
Equal to or more 38	125 (81.7)	28 (18.3)	153 (47.9)		Ref.	
NI *	3	3	6			
Currently have a sexual partner						
No	56 (80)	14 (20)	70 (21.5)	0.84	0.44; 1.63	0.624
Yes	197 (77.3)	58 (22.7)	255 (78.5)		Ref.	
History of Sexually Transmitted Infections in the last six months						
Yes	230 (78)	65 (22)	295 (90.8)	0.92	0.38; 2.26	0.87
No	23 (76.7)	7 (23.3)	30 (9.2)		Ref.	
Sex with more than one partner in the last 6 months						
Yes	41 (71.9)	16 (28.1)	57 (17.8)	1.48	0.77; 2.83	0.23
No	209 (79.2)	55 (20.8)	264 (82.2)		Ref.	
NI *	3	1	4			
Condom use during recent sexual intercourse						
No	177 (80.8)	42 (19.2)	219 (68.7)	0.58	0.33; 1.00	0.05
Yes	71 (71)	29 (29)	100 (31.3)		Ref.	
NI *	5	1	6			
Condom broken during sexual intercourse						
Yes	56 (67.5)	27 (32.5)	83 (26.3)	2.0	1.14; 3.51	0.01
No	187 (80.6)	45 (19.4)	232 (73.7)		Ref.	
NI *	10	0	10			
Use of alcohol and/or other drugs before sexual intercourse						
Yes	87 (76.3)	27 (23.7)	114 (35.7)	1.1	0.64; 1.90	0.72
No	160 (78)	45 (22)	205 (64.3)		Ref.	
NI *	6	0	6			
Knows the HIV serology of the sexual partners						
No	185 (78.7)	50 (21.3)	235 (72.3)	0.83	0.47; 1.48	0.53
Yes	68 (75.6)	22 (24.4)	90 (27.7)		Ref.	

Legend: * NI: not informed–not considered for statistical calculation. OR: odds ratio. CI: confidence intervals. Ref.: reference.

**Table 3:** Programmatic factors associated with serology for *C. trachomatis* among riverine, in the Brazilian Amazon, 2020–2021.

PROGRAMMATIC	Non-Reagent	Reagent	Total	Binary Regression		
	*n* (%)	*n* (%)	*n* (%)	Crude OR	*n* (%)	*n* (%)
Performed rapid test for Sexually Transmitted Infections—ever in life						
No	119 (81.5)	27 (18.5)	146 (46.9)	0.66	0.38; 1.14	0.14
Yes	123 (74.5)	42 (25.5)	165 (53.1)		Ref.	
NI *	11	3	14			
Performed rapid test for HIV—In the last 12 months						
No	182 (80.5)	44 (19.5)	226 (72.9)	0.60	0.33; 1.07	0.08
Yes	60 (71.4)	24 (28.6)	84 (27.1)		Ref.	
NI *	11	4	15			
Performed rapid test for syphilis						
No	130 (82.8)	27 (17.2)	157 (50.3)	0.55	0.32; 0.96	0.03
Yes	113 (72.9)	42 (27.1)	155 (49.7)		Ref.	
NI *	10	3	13			
Free access to condoms in the last 12 months						
No	157 (82.2)	34 (17.8)	191 (59.9)	0.51	0.30; 0.87	0.01
Yes	90 (70.3)	38 (29.7)	128 (40.1)		Ref.	
NI *	6	0	6			
Free access to female condom in the last 12 months						
No	226 (78.2)	63 (21.8)	289 (88.9)	0.83	0.37; 1.86	0.66
Yes	27 (75.0)	9 (25.0)	36 (11.1)		Ref.	
Know Post-Exposure Prophylaxis						
I just heard	12 (66.7)	6 (33.3)	18 (5.5)	2.0	0.31; 12.5	0.45
No	233 (78.5)	64 (21.5)	297 (91.4)	1.09	0.22; 5.30	0.90
Yes	8 (80)	2 (20)	10 (3.1)		Ref.	

Legend: * NI: not informed–not considered for statistical calculation. ORc: odds ratio crude. CI: confidence intervals. Ref.: reference.

**Table 4:** Results of the multiple logistic regression analysis between factors and *C. trachomatis* among riverine, in the Brazilian Amazon, 2020–2021.

Factos	OR Adjusted	95% CI	*p*-Value
Number of people living in the house (Ref.: 1 to 2)			
Equal to 3 or more	1.49	(0.72; 3.10)	0.27
Participate in social programs (Ref.: No)			
Yes	1.44	(0.73; 2.83)	0.28
Sex (Ref.: Female)			
Male	0.58	(0.30; 1.11)	0.10
Condom use during recent sexual intercourse (Ref.: Yes)			
No	0.75	(0.39; 1.42)	0.38
Condom broken during sexual intercourse (Ref.: No)			
Yes	1.90	(1.01; 3.57)	0.04
Performed rapid test for Sexually Transmitted Infections—ever in life (Ref.: Yes)			
No	1.02	(0.46; 2.24)	0.94
Performed rapid test for HIV—In the last 12 months (Ref.: Yes)			
No	1.05	(0.48; 2.28)	0.89
Performed rapid test for HIV (Ref.: Yes)			
No	0.78	(0.37; 1.64)	0.51
Free access to condoms in the last 12 months (Ref.: Yes)			
No	0.62	(0.33; 1.14)	0.12

Legend: OR: odds ratio. CI: confidence intervals. Ref.: reference.

## Data Availability

The original contributions presented in the study are included in the article, further inquiries can be directed to the corresponding author at glendaf@ufpa.br.
